# Importance of Leadership Style towards Quality of Care Measures in Healthcare Settings: A Systematic Review

**DOI:** 10.3390/healthcare5040073

**Published:** 2017-10-14

**Authors:** Danae F. Sfantou, Aggelos Laliotis, Athina E. Patelarou, Dimitra Sifaki- Pistolla, Michail Matalliotakis, Evridiki Patelarou

**Affiliations:** 1 2nd Department of Cardiology, Attikon University Hospital, National and Kapodistrian University of Athens Medical School, Athens 12462, Greece; danaes230@gmail.com; 2 Department of Upper Gastrointestinal and Bariatric Surgery, St. Georges, NHS Foundation Hospitals, London SE170QT, UK; laliotisac@gmail.com; 3 Department of Anesthesiology, University Hospital of Heraklion, Crete 71500, Greece; athina.patelarou@gmail.com; 4 Clinic of Social and Family Medicine, School of Medicine, University of Crete, Crete 71500, Greece; spdimi11@gmail.com; 5 Department of Obstretics and Gynaecology, Venizeleio General Hospital, Heraklion, 71409, Greece; mihalismat@hotmail.com; 6 Florence Nightingale Faculty of Nursing and Midwifery, King’s College, London SE18WA, UK

**Keywords:** leadership, leadership style, quality of care, nursing

## Abstract

Effective leadership of healthcare professionals is critical for strengthening quality and integration of care. This study aimed to assess whether there exist an association between different leadership styles and healthcare quality measures. The search was performed in the Medline (National Library of Medicine, PubMed interface) and EMBASE databases for the time period 2004–2015. The research question that guided this review was posed as: “Is there any relationship between leadership style in healthcare settings and quality of care?” Eighteen articles were found relevant to our research question. Leadership styles were found to be strongly correlated with quality care and associated measures. Leadership was considered a core element for a well-coordinated and integrated provision of care, both from the patients and healthcare professionals.

## 1. Introduction

Nowadays, both evidence-based medicine and nursing are widely recognized as the tools for establishing effective healthcare organizations of high productivity and quality of care. Management and leadership of healthcare professionals is critical for strengthening quality and integration of care. Leadership has been defined as the relationship between the individual/s who lead and those who take the choice to follow, while it refers to the behaviour of directing and coordinating the activities of a team or group of people towards a common goal [1,2]. There are many identified styles of leadership, while six types appear to be more common: transformational, transactional, autocratic, laissez-faire, task-oriented, and relationship-oriented leadership. Transformational leadership style is characterized by creating relationships and motivation among staff members. Transformational leaders typically have the ability to inspire confidence, staff respect and they communicate loyalty through a shared vision, resulting in increased productivity, strengthen employee morale, and job satisfaction [3,4]. In transactional leadership the leader acts as a manager of change, making exchanges with employees that lead to an improvement in production [3]. An autocratic leadership style is considered ideal in emergencies situation as the leader makes all decisions without taking into account the opinion of staff. Moreover, mistakes are not tolerated within the blame put on individuals. In contrary, the laissez-faire leadership style involves a leader who does not make decisions, staff acts without direction or supervision but there is a hands-off approach resulting in rare changes [4]. Task-oriented leadership style involves planning of work activities, clarification of roles within a team or a group of people, objectives set as well as the continuing monitoring and performance of processes. Lastly, relationship-oriented leadership style incorporates support, development and recognition [5].

Quality of care is a vital element for achieving high productivity levels within healthcare organizations, and is defined as the degree to which the probability of achieving the expected health outcomes is increased and in line with updated professional knowledge and skills within health services [6]. The Institute of Medicine OM has described six characteristics of high-quality care that must be: (1) safe, (2) effective, (3) reliable, (4) patient-centred, (5) efficient, and (6) equitable. Measuring health outcomes is a core component of assessing quality of care. Quality measures are: structure, process, outcome, and patient satisfaction [6]. According to the National Quality Measures Clearing House (USA), a clinical outcome refers to the health state of a patient resulting from healthcare. Measures on patient outcomes and satisfaction constitute: shorter patient length of stay, hospital mortality level, health care-associated infections, failure to rescue ratio, restraint use, medication errors, inadequate pain management, pressure ulcers rate, patient fall rate, falls with injury, medical errors, and urinary tract infections [7].

There are numerous publications recognizing leadership style as a key element for quality of healthcare. Effective leadership is among the most critical components that lead an organization to effective and successful outcomes. Significant positive associations between effective styles of leadership and high levels of patient satisfaction and reduction of adverse effects have been reported [8]. Furthermore, several studies have stressed the importance of leadership style for quality of healthcare provision in nursing homes [9]. Transformational leadership is strongly related to the implementation of effective management that establishes a culture of patient safety [10]. In addition, the literature stresses that empowering leadership is related to patient outcomes by promoting greater nursing expertise through increased staff stability, and reduced turnout [11]. Effective leadership has an indirect impact on reducing mortality rates, by inspiring, retaining and supporting experienced staff. Although there are many published studies that indicate the importance of leadership, few of these studies have attempted to correlate a certain leadership style with patient outcomes and healthcare quality indicators. 

Therefore, the aim of this review was to identify the association between leadership styles with healthcare quality measures.

## 2. Materials and Methods

This systematic review was designed and conducted in line with the published guidelines for reporting systematic reviews and meta-analyses [12]. Systematic review of the existing literature on leadership style and quality of healthcare provision was performed. The main review question was: “Which is the relationship between styles of leadership in healthcare settings and quality of care?” A systematic, comprehensive bibliographic search was carried out in the National Library of Medicine (Medline) and EMBASE databases for the time period between 2004–2015 in the PubMed interface. Search terms used were chosen from the USNML Institutes of Health list of Medical Subject Headings (MeSH) for 2015. The included MeSH terms were: “Nurse Administrators”; “Nurse Executives”; “Physician Executives”; “Leaders”; “Leadership”; “Managers”; “Management style”; “Leadership style”; “Organizational style”; “Organizational culture/climate”; “Leadership Effectiveness”; “Quality of healthcare”; “Patient outcome Assessment”; “Quality indicators, Healthcare”; “Healthcare quality, Access and Evaluation”; and “Quality Assurance, Healthcare”. References used by each identified study were also checked and included in the study according to the eligibility criteria.

Five major inclusion criteria were adopted:

- Papers published in peer-reviewed journal
- Papers written in the English language
- Papers published from 2004 to 2015 (focus on more recent knowledge)
- Human epidemiological studies
- Studies used a quantitative methodology reporting the leadership style and healthcare quality measures

Studies that did not meet the above criteria were excluded, while those that complied with the inclusion criteria were listed and further reviewed.

Studies were evaluated and critically appraised (Aveyard critical appraisal tool) by two independent reviewers. Literature screening (a three-stage approach-exclusion by reading the title, the abstract, and the full text) and extraction of the data were conducted by two reviewers, independently. In cases of uncertainty, a discussion was held among the members of the team to reach a common consensus. Data were extracted systematically from each retrieved study, using a predesigned standard data collection form (extraction table). The following information was extracted from each one of the included studies (Table 1): authors, year of conduction, country, study design, subjects, population, research purpose, leadership style definition, outcome definition, and main findings.

## 3. Results

### 3.1. Bibliographic Search

**Figure 1:**
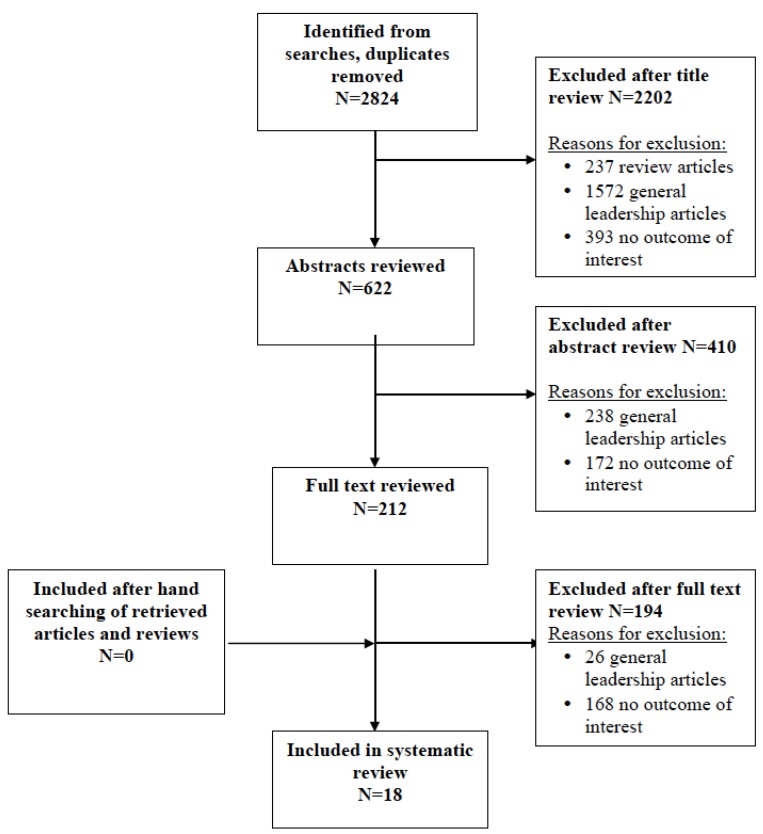
Prisma flowchart.

A total of 2824 records were retrieved through our searches in Medline and EMBASE databases. Following reading the titles and abstracts of the retrieved records 212 remained for further evaluation. Another 194 articles were excluded after reading the full article. Figure 1 shows the exact sequence and process of study identification, selection and exclusion in each step of the search. Finally, 18 studies were considered to be appropriate for answering our primary research question.

### 3.2. Overview of the Included Studies

Among 18 included studies, seven were conducted in the USA, six in Canada, two in Finland, one in Saudi Arabia, one in Kuwait, and one in Norway. Among the relevant studies, 14 were cross-sectional, two were descriptive correlation studies, one was a secondary analysis of data, and one was an exploratory investigation. Diverse care settings were represented in the studies. Identified settings included: hospitals/healthcare settings (*n* = 16), acute and critical care units (*n* = 1), and oncology settings (*n* = 1). In addition, study samples consisted exclusively of employees (*n* = 16), or combination of employees and managers (*n* = 2). Patient safety climate, patient satisfaction, mortality, and quality of care were the main outcomes of interest. Leadership was assessed in these studies according to leadership styles, behaviors, perceptions, and practices. The most commonly used tool to measure leadership was the Multifactor Leadership Questionnaire, MLQ, (*n* = 7). The variety of the quality measures and different definitions/scales used among a limited number of included studies did not allow the performance of a meta-analysis of the retrieved findings.

### 3.3. Leadership Style and Patients Outcomes

Improved quality of healthcare services (moderate-severe pain, physical restraint use, high-risk residents having pressure ulcers, catheter in bladder) was reported for consensus manager leadership style [28]. Resonant leadership influenced the quality of safety climate which, in turn, impacted on medication errors [27]. Resonant leadership style was related to lower 30-day mortality and presented a strong association of 28% lower probability of 30-day mortality comparing with high-dissonant (14% lower) followed by hospitals with mixed leadership styles [24]. The task-oriented leadership style was found to relate to higher levels of quality of care based on the assessment made by relatives and staff [9]. Furthermore, formal leadership style was positively associated with learning from minor and moderate patient safety events, while informal leadership presented no effect [25]. Patients were more satisfied when the manager followed a transactional leadership style [24]. However, Raup found that there was no association between leadership style and patient satisfaction [19].

### 3.4. Organizational Culture and Quality of Care

Important relationships between workplace enforcement and practice environmental conditions for staff nurses and patient safety were observed [14]. Authentic hands-on leadership style, behaviors and organizational practices of distinctive leadership were associated with significant differences in patient level measure of quality and safety; such as mortality patterns, patient safety, equity and effectiveness in care [15]. Transformational leadership was found to positively relate with effective nursing unit organization culture, while transactional leadership had a weak relationship. In addition, laissez-faire leadership was negatively related to nursing unit organization culture [18]. Findings confirmed that the higher total structural empowerment score was correlated to a higher safety level and empowering workplaces contributed to positive effects on nursing quality of care [23,26]. Higher entrepreneurial culture was also related to higher levels of safety climate for the patient [30]. Alahmadi also found that the variables that contributed to patient safety score included management role, organization learning, continuous improvement, communication, teamwork, and feedback about errors [22]. Singer et al. found that higher group culture was associated with higher safety climate overall but more hierarchical culture was correlated with lower safety climate suggesting that general organizational culture is important to organizations’ climate of safety [21]. Role ambiguity and role conflict on the units were found to relate to higher turnover rates for nurses. The increased likelihood of medical error was related to the higher level of role ambiguity and a higher turnover rate. Finally, lack of employer care and team support were the most common reasons for leaving [31].

## 4. Discussion

Effective leadership in health services has already been extensively studied in the literature, especially during the last decades [32]. Several societal challenges have revealed the urgent need for effective leadership styles in health and social services. Nevertheless, studies that use quantitative data or assess the impact of leadership in health care quality measures are neglected, while most studies have adopted a qualitative approach [33]. The present literature review attempted to fill this gap, while it managed to identify the most recent publications to assess the correlation between leadership styles with healthcare quality measures. 

Among the main findings, correlation of leadership with quality care and a wide range of patient outcomes (e.g., 30-day mortality, safety, injuries, satisfaction, physical restraint use, pain, etc.) were stressed in most of the identified articles [9,24,27,28]. Therefore, leadership is considered a core element for a well-coordinated and integrated provision of care, both from the patients and healthcare professionals. It is essential regardless of where care is delivered (e.g., clinics or inpatient units, long-term care units, or home care facilities), especially for those who are directly involved with patients for long periods of time [34].

Additionally, effects of leadership style on patient outcomes were evident in the aforementioned findings. Other studies [35] agree with our main findings and stress the theoretical interactions of effective leadership and patient outcome as follow; effective leadership fosters a high-quality work environment leading to positive safety climate that assures positive patient outcomes. Failure of leadership to create a quality work place ultimately harms patients [29,35]. Most of these studies are focusing on nursing leadership. Particularly, as also reported by the current study, transformational and resonant leadership styles are associated with lower patient mortality, while relational and task-oriented leadership are significantly related to higher patient satisfaction [35,36,37]. Furthermore, increased patient satisfaction in acute care and homecare settings has been found to be closely related to transformational, transactional, and collaborative leadership [36,37]. Overall, the vast majority of studies assessing patient outcomes in the literature, have reported adverse outcomes defined as unintentional injuries or complications associated with clinical management, rather than the patient’s primary condition, resulting in death, disability, or extended stay in hospital [17,37]. 

Furthermore, leadership has been recognized as a major indicator for developing qualitative organizational culture and effective performance in health care provision [14]. Similarly to our study, other studies that used primary quantitative data revealed a strong correlation of leadership and safety, effectiveness, and equity in care. For instance, transformational leadership increases nursing unit organization culture and structural empowerment [18]. This has an impact on organizational commitment for nurses and in return higher levels of job satisfaction, higher productivity, nursing retention, patient safety, and overall safety climate, and positive health outcomes [18,23,38]. In addition, safety climate was among the main findings of our study. As supported by the literature [38], a safety climate connected to transformational leadership style is strongly linked to improved process quality, high organization culture and positive patient outcomes. Therefore, safety climate is directly associated with improved patient safety outcomes and the overall quality of care. 

The literature has identified the significance of leadership styles and practices on patient outcomes, health care workforce and organizational culture. Setting effective leadership as a priority in health care units is expected to enhance a variety of measurable indicators, even in fragmented health systems [39]. Nowadays, more and more regional and national health systems tend to undergo structural changes and redesign their functions and priorities in order to face modern societal, economic, and health challenges and needs [17]. Medical leadership in decision-making is a key component in order to develop a successful and qualitative priority setting process in health care. Most importantly, engagement of non-medical clinical leaders, such as nursing leadership, is considered to ensure the legitimacy and validity of priority setting [40]. As shown in the present study, the leadership styles that proved to be more effective and promoted positive outcomes were those that conceptualize management as a collaborative, multifaceted, and dynamic process (e.g., transformational, employee-oriented leadership). 

Future research has to focus on the development, feasibility and implementation of robust leadership styles models in diverse health care settings. These studies should include multidisciplinary professional teams, strengthen the role of nurses and other health care professionals, explore additional quality of life and healthy ageing indicators (both for professionals and patients), and address organizational parameters and individual wishes, preferences, and expectations towards quality in health care [17,37,40,41,42,43,44].

## 5. Conclusions

Leadership styles play an integral role in enhancing quality measures in health care and nursing. Impact on health-related outcomes differs according to the different leadership styles, while they may broaden or close the existing gap in health care. Addressing the leadership gap in health care in an evolving and challenging environment constitutes the current and future goal of all societies. Health care organizations need to ensure technical and professional expertise, build capacity, and organizational culture, and balance leadership priorities and existing skills in order to improve quality indicators in health care and move a step forward. Interpretation of the current review’s outcomes and translation of the main messages into implementation practices in health care and nursing settings is strongly suggested.

## Figures and Tables

**Table 1:** An overview of studies’ characteristics, outcome definitions and main findings.

Author et al. (year)	Main Study Characteristics	Aim of the Study	Leadership Style Definition	Outcome Definition	Main Findings
Al-Mailam (2004) [13]	Kuwait, cross-sectional study Four public and private hospitals 266 administrators and physicians	To explore the impact of leadership styles on employee perception of leadership efficacy.	Two categories of administrators’ and physicians’ leadership style: - Transformational leaders - Transactional leaders	Leadership style (Multifactor Leadership Questionnaire)	**Leadership style** (midpoint = 33, average score) Hospital director: 26.89 Department Head: 25.74 **Leadership efficacy** [midpoint = 6.0 average score, (F-value)] Both Medical director and Department Head = 4.44, (32.41 and 48.43) **Type of hospital and transformational leadership style** (average score, (SE)) public vs. private hospital Hospital director: 29.48 (0.71) vs. 24.62 (0.73) Department head: 27.28 (0.71) vs. 24.41 (0.67)
Armstrong et al. (2006) [14]	Central Canada, Small community hospital 40 staff nurses	To test a theoretical model.		**Structural empowerment** *(Conditions of Work Effectiveness Questionnaire-II)* **Magnet hospital characteristics—Practice Environment** *(Lake’s Practice Environment Scale of the Nursing Work Index, PES of NWI)* **Safety climate** *(The Safety Climate Survey)*	**Total Empowerment scale** [mean score (SD)] 17.1 (4.26) Cronbach α = 0.94 **Total PES** [mean score(SD)] 2.5 (0.64) Cronbach α = 0.85 **Safety Climate** [mean score(SD)] 3.53 (0.80) Cronbach α = 0.81 **Empowerment and professional practice characteristics** [r (*p*-value)] Nursing model of care 0.61 (<0.01) Management ability 0.52 (<0.01) Collaborative relationships 0.316 (<0.005) **Empowerment and patient safety culture** [r (*p*-value)] Patient safety culture 0.50 (<0.01) Support 0.51 (<0.01) Informal power 0.43 (<0.01) Opportunity 0.45 (<0.01) **Combined effect of magnet hospital characteristics on patient safety culture and empowerment** 46% of variance, F = 13.32, dF = 1.31 *p* = 0.0001
Keroack et al. (2007) [15]	US, 2003–2005 Exploratory investigation 79 Academic Medical Centers patient-level data site visits	To identify organizational factors associated with quality and safety performance.	Hospitals’ leadership style: - Authentic hands-on leadership style	**Patient safety** *(Agency for health* Care Research and Quality, AHRQ-preventable complications, and Patient Safety Indicators) **Mortality** *(mortality rates bases on AHRQ and inpatient quality indicators, IQIs)* **Effectiveness** *(The Joint Commission Hospital Core Measures)* **Equity** *(Measures)*	Composite scores for quality and safety CI 95% (median score %) Group 1 vs. Group 2 vs. Group 3 vs. Group 4 vs. Group 5 67.18% vs. 62.36% vs. 60.22% vs. 58.68% vs. 56.05% **Factors associated with top performing organizations:** Shared sense of purpose Authentic hands-on leadership style Accountability system of quality and safety Focus on results Culture collaboration
Kvist et al. (2007) [16]	Finland Kuopio University Hospital 631 patients 690 nurses 76 managers 128 doctors	To investigate the perception of the quality of care and the relationships between organizational factors and quality of care.		**Quality of care** *(measured by Humane Caring Scale)* **Organizational factors** *(by using questionnaires)*	**Quality of care** (ratings) Patients 1.51 to1.66 Nurses 1.81 to2.19 Managers 1.82 to 2.08 **Organizational factors an Quality of care** - (coefficient of determination)Nursing staff vs. managers vs. physicians0.462 vs. 0.548 vs. 0.337 - [standardized coefficient SC, (*p*-value)] Nursing staff: work vs. values 0.248 (0.01) vs. 0.447 (0.001) Managers: Work vs. leadership 0.472 (0.05) vs. 0.568 (0.05 Physicians: work vs. values 0.289 (0.05) vs. 0.539 (0.05)
Vogus, Sutcliffe (2007) [17]	US, 2003–2004 cross-sectiona l1033 RNs 78 nursing managers 78 care units	To examine the benefits of bundling safety organizing with leadership and design factors on reported medication errors.		**Safety organizing** *(Safety organizing Scale)* ***Trust in manager*** *(2 survey items assessing perceptions for nurse manager)* **Use of care pathways** *(Seven-point Likert Scale, single survey item)* **Reported Medications errors** *(number of errors reported to a unit's incident reporting system)*	**Medications errors** (mean, SD) 12.04, 11.31 **Safety organizing and trusted leadership** (β, coefficient, *p*-value) −0.60, 0.18, *p* < 0.001 **Safety organizing and care pathways** −0.82, 0.25, *p* < 0.001
Casida, Pinto-Zipp (2008) [18]	New Jersey, US, 2006 Four acute care hospitals 37 Nurse Managers 278 staff nurses	To explore the relationship between nursing leadership styles and organizational culture.	Three categories of nurse managers’ leadership style: - Transformational leaders - Transactional leaders - Non-transactional laissez-faire leaders	**Leadership style** *(Multifactor Leadership Questionnaire)* **Nursing unit Organizational culture** *(the Denison’s Organizational Culture Survey)*	**Leadership style** [MLQ scores, mean (SD)] Transformational vs. transactional vs. laissez-faire 2.8 (0.83) vs. 2.1 (0.47) vs. 0.83 (0.90) **NMs’ leadership style and organizational culture** (r, *p*-value) Transformational vs. transactional vs. laissez-faire 0.60 (*p* = 0.00) vs.0.16 *p* = 0.006) vs.−0.34 (*p* = 0.000)
Raup (2008) [19]	US 15 academic health centers 15 managers 15 staff nurses	To explore the role of leadership styles used by nurse managers in nursing turnover and patient satisfaction.	Two categories of ED nurse managers’ leadership style: - Transformational leadersNon - Non-transformational leaders	**Leadership style** *(Multifactor Leadership Questionnaire, MLQ)* **Nurse staff turnover and patient satisfaction** *(managers’ data for nurse turnover and patient safety scores)*	**Leadership style** (% ED nurse managers) transformational vs. Non-transformational 80% vs. 20% **Nurse staff turnover and patient satisfaction** [impact of leadership style: Fisher’s exact test = 0.569] **Mean staff nurse turnover** (%) transformational vs. Non-transformational 13% vs. 29% **Mean ED overall patient satisfaction** (%) transformational vs. Non-transformational76.68% vs. 76.50%
McCutcheon et al. (2009) [20]	Canada Correlation survey Seven hospitals 51 units 41 nurse managers 717 nurses 680 patients	To assess the relationship between leadership style, nurses’ job satisfaction, span of control, and patient satisfaction.	Four categories of managers’ leadership style: - Transformational leaders - Transactional leaders - Management by exception - Laissez-faire	**Nurses**’ **Job Satisfaction** *(measured by McCloskey-Mueller Satisfaction Scale* ***Patient Satisfaction*** (measured by the Patient Judgments of Hospital Quality Questionnaire)	**Nurses**’ **Job Satisfaction** (Mean) 3.2 ***Patient Satisfaction*** (mean) 2.16 (moderate satisfaction) **JS and leadership style** Transformational vs. transactional vs. management by exception vs. laissez-faire (Beta) 0.20 vs. 0.12 vs. −0.08 vs. 0.02 **Span of control and leadership style on JS** Transformational vs. transactional vs. management by exception vs. laissez-faire [coefficient, (*p*-value)] −0.0024 (<0.01) vs. −0.0015 (<0.05) vs. 0.0026 (<0.01) vs. 0.0014 (<0.05) **Span of control and leadership style on patient satisfaction** [coefficient, (*p*-value)] Transformational vs. transactional vs. management by exception vs. laissez-faire −0079(<0.05) vs. −0070 vs. −0103 vs. 0.0045
Singer et al. (2009) [21]	US, 2004–2005 92 hospitals senior managers, physicians, hospital workers questionnaires 18361 safety climate surveys 5637 organizational culture surveys	To assess the aspects of general organizational culture that are related to hospital patient safety climate.		**Safety climate** *(Patient Safety Climate in Healthcare Organization)* **Organizational culture** *(Competing Values Framework)*	**Organisational culture** (average score) hierarchical organizational culture vs. entrepreneurial culture 31.6 points vs. 15.7points **Safety climate** (% PPR-percent problematic response) (higher PPR relates to lower level of safety climate) 17.1% PPR Highest safety climate hospitals vs. lowest safety climate hospitals (mean PPR, *p* = 0.000) 11.5 vs. 24.6 **Relationship of organizational characteristics with patient safety climate** [overall average PPR (SD) *p* < 0.05] group culture vs. entrepreneurial culture vs. hierarchical culture vs. production-oriented culture −0.241 (0.011) vs.−0.279 (0.0022) vs. 0.300 (0.011) vs. 0.0666 (0.017) **Organizational culture and safety climate** [mean (SD] high vs. low safety climate group culture: 40.1 (6.7) vs. 26.9 (7.8) entrepreneurial: 15.3 (2.31) vs. 13.9 (0.9) production-oriented: 20.20 (2.1) vs. 22.4 (2.1) hierarchical: 24.6 (2.8) vs. 36.7 (6.2)
Alahmadi (2010) [22]	Saudi Arabia, 13 general hospitals 223 health professions (nurses, technicians, managers, medical staff)	To assess whether organisation culture supports patient safety.		Patient safety culture *(Hospital Survey on Patient Safety Culture questionnaire)*	**Patient safety** Excellent or very good vs. acceptable vs. failing or poor (%) 60% vs. 33% vs. 7% **Determinants of overall patient safety score**(Standardised coefficient B) Organisational learning/continuous improvement: 0.128 Management role: 0.216 Communication and feedback about errors: 0.215 Teamwork: 0.160
Armellino et al. (2010) [23]	US descriptive correlation study Adult Critical Care Unit (ACCU) tertiary hospital 102 Registered Nurses	To explore the association between structural empowerment and patient safety culture among nurses.		**Structural empowerment, SE** *(Conditions of Workplace Effectiveness Questionnaire)* **Patient safety climate** *(Hospital Survey on Patient Safety Culture)*	**Total structural empowerment, SE** (CWEQ-II, mean score) 20.55 (moderate), Cronbach’s α = 0.89 Moderate SE vs. low level of SE vs. high level of SE (%) 79.2% vs. 1.98% vs. 18.91% **Structural empowerment and patient safety climate (PSC)** - Total CWEQ-II score and overall perception of safety(Pearson’s correlation coefficient)0.32 *p* < 0.05 - Total CWEQ-II empowerment score and HSOPC safety grade(total SE score) Grade A vs. Grade B vs. Grade C vs. Grade D22.667 vs. 20.987 vs. 19.763 vs. 15.889
Cummings et al. (2010) [24]	Canada, 1998–1999 Secondary analysis of data 90 hospitals 21,570 patients 5228 nurses	To explore the association of the role of hospital nursing leadership styles with 30-day mortality.	Five categories of hospitals’ leadership style: - high resonant - moderately resonant - mixed - moderately dissonant - high dissonant	30-day mortality	**Hospital Nursing leadership styles and 30-day mortality** *High dissonant* vs. *moderately dissonant* vs. *mixed type* vs. *moderately resonant* vs. *high resonant (%)* 4.3 vs. 8.8 vs. 8.1 vs. 7.4 vs. 5.2 *High dissonant* vs. *moderately dissonant* vs. *mixed type* vs. *moderately resonant* vs. *high resonant Beta (SE)* Ref vs.−0.64 (0.24) * vs. 0.05 (0.11) vs.−0.08 (0.10) vs.−0.40 (0.19) * *High dissonant* vs. *moderately dissonant* vs. *mixed type* vs. *moderately resonant* vs. *high resonant aOR 95% CI* Ref vs. 0.86 (0.56–1.31) vs. 1.10 (0.96–1.27) vs. 0.90 (0.77–1.04) vs. 0.77 (0.59–1.01)
Ginsburg et al. (2010) [25]	Canada, 2006 Two cross-sectional surveys 49 general acute care hospitals 54 patient safety officers (PSOs) 282 patient care managers (PCMs) PSOs and PCMs questionnaires	To explore organizational leadership towards patient safety and its relationship with five types of learning from patient safety events.	Two categories of organizational leadership style: - Informal organizational - Formal organizational	**Leadership style** *(PCM questionnaire)* **Learning from PSEs** *(four types of PSE-minor/moderate/major events/major near-miss)*	**Learning from PSEs** [Mean (SD)] major event analysis 3.63 (0.56) major event dissemination/communication 2.86 (0.80) moderate event learning 3.03 (0.76) minor events learning 2.53 (0.67) major near-miss events learning 3.03 (0.75)formal organizational leadership 3.90 (0.44) informal organizational leadership 2.34 (1.28) **Learning from Near-miss Events** (β, *p*-value) hospital size −0.339 *p* < 0.10 formal leadership style 0.467 *p* < 0.05 **Learning from Major events dissemination/communication** (β, *p*-value) hospital size and formal leadership style −1.106, *p* < 0.001
Purdy et al. (2010) [26]	Canada, Cross-sectional study 21 hospitals (61 medical and surgery units) 697 nurses 1005 patients	To assess the relationship of nurses' perceptions on their work environment and quality outcomes.		**Work environment** *(Conditions of Workplace Effectiveness Questionnaire, and Work Group Characteristics Measure)* **Patient care quality/patient satisfaction** *(Nursing Care Quality Questionnaire and The Therapeutic Self-care Questionnaire-Acute Care Version)*	**Work environment and patient outcomes** [χ^2^ = 21.074 df = 10] **Work unit** (β, *p*-value) structure empowerment and group processes 0.64 *p* < 0.001 group processes and nurse-assessed quality 0.61 *p* < 0.001 group processes and falls −0.19 *p* < 0.05 group processes and nurse-assessed risk −0.17 *p* < 0.05 **Individual** (β, *p*-value) psychological empowerment and empowerment behavior 0.47 *p* < 0.001 psychological empowerment and job satisfaction 0.39 *p* < 0.001 psychological empowerment and nurse assessed quality of care 0.22 *p* < 0.001
Squires et al. (2010) [27]	Ontario, Canada, 2008 cross-sectiona l267 nurses	To test a model of examining relationships among leadership, interactional justice, work environment, safety climate quality of the nursing and patient and nurse safety.	Nurse managers leadership: - Resonant Leadership	**Leadership** (*measured by Resonant leadership Scale)* **Nursing work environment** *(by using Perceived nursing work environment)* **Safety climate** *(measured by Safety Climate Survey)*	**Final model** χ^2^ = 217.6(138) *p* < 0.001 -resonant leadership and leader-nurse relationship (standardized coefficient) 0.52 nurse leader-nurse relationship and safety climate (standardized coefficient) 0.53 work environment and emotional exhaustion (standardized coefficient) −0.51 safety climate and medication errors (standardized coefficient) −0.22
Castle, Decker (2011) [28]	US, 2008 3867 NHAs (Nursing Home Administrator) 3867 DONs (Director of Nursing)	To assess the relationship of leadership style and quality of care.	Four groups of leaders: - Consensus manager - Consultative autocrat - Shareholder manager - Autocrat	**Leadership style** *(Bonoma-Slevin leadership model)* **Quality of care** *(Nursing Home Compare Quality Measures and 5-Star Rating Scores)*	**Leadership style** Consensus manager vs. consultative vs. shareholder manager vs. autocrat: NHA: 33% vs. 22% vs.19% vs. 26% DON: 30% vs. 20% vs.25% vs. 25% **Leadership and quality of care** [Incident-rate ratio (SE), *p*-value] NHA/DON both Consensus Managers: Percent physical restraint use: 0.97 (0.43), *p* < 0.05 Percent with moderate to severe pain: 0.51 (0.21), *p* < 0.01 Percent high-risk residents with pressure ulcers: 0.62 (0.24), *p* < 0.05 Percent had a catheter inserted and left in bladder: 0.79 (0.19), *p* < 0.001 NHA/DON both Consensus Managers: (Five-star quality measure score, squares regression) 4.02 *p* < 0.01
Havig et al. (2011) [9]	Norway, Cross-sectional study 40 wards of nursing homes 414 employees 13 nursing home directors40 wards managers 444 staff questionnaires 378 relatives 900 h of field observation	To assess the relationship between ward leaders’ task—and leadership styles, on measures of quality of care.	2 categories of hospitals’ leadership style: - Task-oriented leaders - Relationship-oriented leaders	**Quality of care** *(The national regulation for quality of care in nursing homes and home care)* **Staffing** **Care level**	**Leadership style and quality of care** [coefficient (*p*-value) Task-oriented leadership style Relatives vs. staff vs. field observations 0.36 (0.02) vs. 0.63 (>0.01) vs. 0.28 (0.12) Relationship-oriented leadership style 0.12 (0.19) vs. 0.01 (0.91) vs. 0.10 (0.37) **Staffing and quality of care** [coefficient (*p*-value)Total staffing level Relatives vs. staff vs. field observations −0.95 (0.31) vs. 0.10 (0.90) vs. 1.17 (0.30) Ratio of RNs 0.32 (0.66) vs. 0.52 (0.42) vs. 0.20 (0.83) Ratio of unlicensed staff −2.05 (>0.01 vs. −0.80 (0.22) vs. −2.59 (>0.01) **Care level** [coefficient (*p*-value) Relatives vs. staff vs. field observations −0.20 (>0.01) vs. −0.11 (>0.01) vs. −0.11 (0.02)
Kvist et al. (2013) [29]	Finland, 2008–2009 Cross-sectional, descriptive quantitative design Four hospitals 2566 patients Nursing staff	To examine nurses’ and patients’ perceptions of the Magnet model components of transformational leadership and quality outcomes.	One category of hospitals’ leadership style: - Transformational leadership style	**Transformational Leadership style** *(transformational leadership scale)* **Job satisfaction** *(The Kuopio University Hospital Job Satisfaction)* **Patient Safety Culture** *(The Hospital Survey on Patient Safety Culture)* **Patient Satisfaction** *(Revised Humane Caring Scale)*	**Transformational Leadership style** Support for professional development by nurse managers (mean, SD) 3.66, 0.96 **Patient Safety Culture** (mean, SD)Teamwork within units 3.64, 0.69 Supervision 3.60, 0.80 Communication openness 3.57, 0.68 **Patient Satisfaction** (mean, SD, *p*-value) Professional practice 4.49, 0.67 Human resources 3.80, 1.13 **PS average score** *(mean, SD) 4.18, 0.69* **Total JS** *(mean, SD) 3.59, 0.62* **Transformational leadership** *(mean, SD) 3.47, 0.81* **Patient Safety Culture** *(mean, SD) 3.3, 0.47*
